# Development and evaluation of an anesthesia module for electronic medical records in the operating room: an applied developmental study

**DOI:** 10.1186/s12871-023-02335-2

**Published:** 2023-11-17

**Authors:** Marjan Jokar, Mohammad Ali Sahmeddini, Farid Zand, Rita Rezaee, Azadeh Bashiri

**Affiliations:** 1 Department of Health Information Management, School of Health Management and Information Sciences, Health Human Resources Research Center, Shiraz University of Medical Sciences, Shiraz, Iran; 2 Department of Anesthesiology, School of Medicine, Anesthesiology and Critical Care Research Center, Shiraz University of Medical Sciences, Shiraz, Iran; 3 Department of Health Information Management, School of Health Management and Information Sciences, Health Human Resources Research Center, Clinical Education Research Center, Shiraz University of Medical Sciences, Shiraz, Iran

**Keywords:** Anesthesia module, Electronic medical records, Operating room, Hospital information system

## Abstract

Developing an anesthesia module in the operating room is one of the significant steps toward the implementation of electronic medical records (EMR) in health care centers. This study aimed to develop and evaluate the web based-anesthesia module of an electronic medical record Sciences, in the operating room of the Namazi Medical Training Center of Shiraz University of Medical Iran. This developmental and applied study was conducted in steps including determining the functional and non-functional requirements, designing and implementing the anesthesia module, and usability evaluation. 3 anesthesiologists, 3 anesthesiologist assistants, and 12 anesthetist nurses were included in the study as a research community. React.js, Node.js programming language to program this module, Mongo dB database, and Windows server for data management and USE standard questionnaire were used. In the anesthesia module, software quality features were determined as functional requirements and non-functional requirements included 286 data elements in 25 categories (demographic information, surgery information, laboratory results, patient graphs, consults, consent letter, physical examinations, medication history, family disease records, social record, past medical history, type of anesthesia, anesthesia induction method, airway management, monitoring, anesthesia chart, blood and fluids, blood gases, tourniquets and warmers, accessories, positions, neuromuscular reversal, transfer the patient from the operating room, complications of anesthesia and, seal/ signature). Also, after implementing the anesthesia module, results of the usability evaluation showed that 69.1% of the users agreed with the use of this module in the operating room and considered it user-friendly.

## Introduction

Information technology in the health sector leads to better management of those types of information that healthcare providers need to improve the effectiveness of their duties [1]. The operating room is a black box for many hospital administrators and senior executives. Patients may share common diagnoses and undergo standard surgical procedures but often have different outcomes and costs associated with their care [2]. Recording inconsistent or uncertain information, especially for unusual events, jeopardizes efforts to quantitatively analyze events, understand their consequences, and evaluate corrective actions [3]. Complete and accurate documentation of specialized services is essential for patient care, providing evidence of compliance with basic standards of care, ensuring compliance with regulatory agencies, and accreditation and optimizing reimbursement for services provided [4].

The Electronic Medical Record (EMR) system is considered a significant evolution in providing medical services [5–7] as one of the popular topics in electronic health. Widely used EMR systems can increase the quality of work of health care providers and, subsequently, better patient care. The electronic medical record includes information related to the health of patients and the primary forms of electronic health applications. In addition, the electronic medical record has legal records created in medical centers and outpatient environments [8]. The benefits of electronic medical records include lists of medications, notes, and legible prescriptions, instantly available charts, reduced chart tension, fewer transcription costs, reduced medical errors, and improved quality of care and standards in patient safety [8, 9].

The safety of patients who need anesthesia during surgery has also increased with the advancement of medical engineering and technologies in the field of anesthesia. Anesthesia still has many risks due to various causes, including the error of the medical staff and doctors or high-risk patients. Safe clinical care depends on obtaining and recording complete and timely information before it is forgotten [9]. In the 1890s, Dr. Cadman and Cushing introduced paper documentation of patients' physiological status during anesthesia, which included measurable data such as pulse, temperature, respiratory rate, and blood pressure. With the advent of computers in the 1970s and 1980s, anesthesia providers began to explore electronic recording, storage, retrieval, and formatting of intraoperative data. Duke's automated monitoring system was among the first to record patients' anesthesia data. Today, anesthesia information management systems range from simple record keepers to comprehensive software solutions, and are used in various patient care settings, including operating rooms, delivery rooms, hospitals, acute pain services, outpatient clinics, and intensive care units [10, 11]. The purpose of anesthesia information management systems is to document a patient's response to anesthesia and surgery by recording procedures, physiological changes, key events, and drug administration that occur during the pre-, intra-, and post-operative stages [10]. Anesthesia providers must record every detail of patient care during anesthesia protocol implementation because anesthesia records must be kept meticulously. However, manually documented anesthesia records often lack relevant and valuable information [12].

Managing difficulties during anesthesia is always challenging, and every disorder requires accurate and appropriate information recording. Also, anesthesia service providers in the operating room showed a greater need for electronic anesthesia record data to aid clinical decision-making [13]. Based on recent studies, anesthesia information management systems are specialized electronic health record networks that allow anesthesia records to communicate with hospital clinical data repositories and improve the quality of care, patient safety, operations management, reimbursement, and translational research [13, 14]. Electronic anesthesia records in comparison handwritten anesthesia records, had significantly higher information completion rate which support the increase in document quality and also in evaluation of user satisfaction, had best performance to save time and cost [12]. It allows centers to select appropriate quality improvement initiatives, support patient-centered and improvement-centered practices, and deliver at the clinical, organizational, and community levels [15]. Implementing the anesthesia module in the electronic health record improved the analytical process, highlighting better patient identification and registration, fewer programming or moving errors, and shorter response times [16, 17].

Information and communication technology can solve problems in clinical departments, including managing large volumes of data and reducing medical errors [18, 19]. In this regard, it seems necessary to use electronic data registration and replace them with the current method to increase the accuracy of anesthesia information and record them in time. Therefore, this research aims to develop and evaluate the web-based anesthesia module in the operating room of Namazi Medical Training Center.

## Material and methods

This research is generally developmental and applied in terms of results. In more detail, the type of study in the first objective is descriptive-library, in the second and third objectives are developmental, and the type of study in the fourth objective is descriptive. The study area is the general operating room of Namazi hospital which is affiliated whit Shiraz University of Medical Sciences. Research community were anesthesiologists, anesthesiologist assistants, and anesthetist nurses. Research sampling was not performed due to the limited size of the research population.

### Determining the functional and non-functional requirements of the anesthesia module of the electronic medical file of the operating room department

The following four steps were carried out to determine the functional and non-functional requirements of the electronic medical record in the operating room department.

In the first stage, the researcher went to the operating room department of Namazi Medical Education Center and checked the existing forms of that center in connection with the registration of anesthesia information and extracted all the information elements recorded for the patients of this department.

In the second step, valid databases and all studies were reviewed, including articles, theses, reports, and guidelines in Farsi and English, without time limits.

In the third step, the researcher integrated the data elements and requirements (functional and non-functional) of the anesthesia module of the electronic medical record in the operating room section resulting from the first and second steps. Further, a complete and separate list of related items was prepared.

### Designing the anesthesia module of the electronic medical record in the operating room section of the Namazi Medical Training Center of Shiraz University of Medical Sciences:

At this stage, the design of the anesthesia module of the electronic medical record in the operating room department was carried out in the following five steps:

#### Designing initial anesthesia form

The researcher designed all the data elements necessary for the anesthesia module of the electronic medical record in the operating room department, which was extracted from objective one, using Word and Photoshop as a five-page form. This form included identity information (demographic) and clinical information.

Identity information includes first name, last name, father's name, date of birth, height, weight, age, nationality, case number, gender, date of admission, date of anesthesia, and department of admission.

Clinical information includes 24 main groups: 1- Surgery information 2-Laboratory results 3-Patient graphs 4-Patient consultations 5-Consent letter 6-Physical examinations 7-Medication history 8-Family disease records 9-Social records 10-Past medical history, 11-Type of anesthesia 12-Anesthesia induction method 13-Airway management 14-Monitoring 15-Anesthesia chart, 16-Blood and fluids 17-Blood gases 18-Tourniquets and warmer 19-Accessories 20-Position 21-neuromascular reversal 22-Transfer patient from the operating room, 23- Complication of anesthesia 24-Seal and signature of the documenting physician and nurse anesthetist.

#### Evaluation of the initial form

The researcher held five focused group discussion sessions (1.5h) with a panel of experts, including three anesthesiologists working at Shiraz University of Medical Sciences and two experts in health information management, to evaluate the components of the initially designed form.

#### The prototype of the form designed in the operating room section of the Namazi therapeutic training center

After designing the prototype of the form, the specialists of the panel of experts agreed that the initial version of the form should be implemented in paper form for one week in the general operating room at the prayer therapy training center of Shiraz University of Medical Sciences. Then, the results are analyzed to ensure the quality of the prototype of the designed form in collecting anesthesia information required by the operating room department.

#### Preparation of data dictionary

A data dictionary is a collection of information about data that effectively helps others understand the meaning of data, describes the main information elements and structure of data, and serves as an index to identify all the data in the system. A data dictionary is a supplementary document that describes the information provided about data in detail and is the primary source of standard definitions for information elements and data structures in the system, including name, substitute, description, size and value, and unit [20].

At this stage, the researcher prepared the data dictionary, approved by the research team, and applied the experts' suggestions. The researcher and the anesthesiologists carefully reviewed the acceptable items for each information element and the acceptable range for each information element in preparing the data dictionary.

#### Scenario writing and review

The research team approved the scenarios prepared by the researcher. According to the experts’ opinion, things like mandatory registration of information about underlying diseases of the person, registration of tracheal tube type and its size, stylet type were added, and things like blind intubation and some laboratory results like AST/ALT, Acidosis, and Alkalosis were deleted. Finally, the scenarios were modified and approved.

### Implementation of the anesthesia module of the electronic medical record in the operating room department of the prayer therapy training center of Shiraz University of Medical Sciences

The implementation of the anesthesia module of the electronic medical record in the operating room department was carried out in three steps:

### Software programming

On November 3, 2022, the proposed plan, along with all the necessary documentation (including the data dictionary, scenarios, and the final sample designed from the paper form), was presented to the software designer team during a single session. The session lasted an average of two hours and was attended by the researcher and two anesthesiologists. After seven months, the programming team developed a prototype of the web application using React.js and Node.js programming languages. The team also used Mongo dB database and Windows server for data management.

#### Teaching the software to users

To train the users, the researcher began by compiling a list of all participants, which included three anesthesiologist assistants and twelve anesthesiologists. Three training sessions were then conducted at the users' workplace, located in the general operating room of Namazi Hospital. Each session lasted an average of two hours and covered instruction on how to use the module. During the training, the researcher was present in the operating room to address any questions or issues that arose while users were working with the system.

#### Aanesthesia module test of electronic medical records in the operating room department of Namazi Medical Training Center

In order to implement this module, two computers were used within the department. The software was then trialed for a week in the operating room department to identify any implementation issues and weaknesses. This trial allowed for the complete implementation of the system with minimal problems and obstacles. Throughout the trial, the researcher recorded any feedback, suggestions, or comments from doctors, assistants, and anesthesia experts. Following the trial, the programming team updated the software and resolved any reported problems or deficiencies.

### Evaluation of the applicability of the anesthesia module of the electronic medical record in the operating room department of the Namazi Medical Training Center of Shiraz University of Medical Sciences

At this stage, the applicability of the anesthesia module of the electronic medical record in the operating room department was evaluated in the following two steps:

#### Distribution of evaluation questionnaire

To evaluate the applicability of the anesthesia module of the electronic medical record in the operating room department, the researcher utilized the USE standard questionnaire, which has been previously validated and tested for reliability [19]. The questionnaire consists of 30 questions, divided into four parts: usefulness (8 questions), ease of use (11 questions), easy learning (4 questions), and satisfaction (7 questions), all rated on a 7-point Likert scale from completely disagree to completely agree. An expert panel determined that 15 users of the anesthesia module in the operating room department would be sufficient for the evaluation. The researcher obtained permissions to distribute the questionnaire and explained the purpose of the evaluation to the users.

#### Analysis of the evaluation results

After distributing and collecting the questionnaires, the analysis of the collected data was done using the statistical software SPSS version 27.

It is worth mentioning that in this research, the medical records of patients were obtained from a teaching hospital where prior patient consent had been obtained for the use of their medical information for clinical and research goals. After the approval of the research ethics committee (IR.SUMS.NUMIMG.REG.1400.024), all the information of this research was collected and kept confidential. After distributing and collecting the questionnaires, the data were analyzed using the statistical SPSS software version 27.

## Results

In this study, three anesthesiologists, three anesthesiologist assistants, and 12 anesthetist nurses were included as research population.

### Functional and non-functional requirements of the anesthesia module of the electronic medical record of the operating room department

The results were presented as the requirements of the anesthesia module of the electronic medical record in two main groups of functional and non-functional requirements. The non-functional requirements of the relevant module were categorized into demographic information elements, clinical information elements and general requirements.

By reviewing anesthesia form in the operating room of Namazi Hospital 70 data elements were identified. Some data elements were unstructured and accessible text, and some were structured. The 15 general categories in this form, were identity information (demographic), hospitalization information, physical assessment of the patient, drugs taken by the patient, doctor, and surgeon information, monitoring before, during, and after anesthesia, anesthesia chart, anesthesia method, pre-anesthesia drugs, blood and fluids, primary and secondary diagnosis, surgery performed and position, anesthesia complications, anesthesia time and surgery, and the signature of the anesthesiologist and anesthetist nurses, each with its sub-categories. Also, by reviewing articles, guidelines and anesthesia forms from the other countries, a total of 242 data elements were obtained (312 data elements in total). After discussion and exchange of opinions in the expert panel, some unnecessary information elements were removed. The final result was 286 approved information elements, which included both identity information (demographic) and clinical information which is equivalent to 90% of the total primary data elements.

Table 1 presents the information elements of the patient's identity. In addition, clinical information elements were categorized into 24 main categories, including (Surgery information, Laboratory results, Patient graphs, consults, Consent letter, Physical examinations, Medication history, Family disease records, Social record, Past medical history, Type of anesthesia, Anesthesia induction method, Airway management, Monitoring, Anesthesia chart, Blood and fluids, Blood gases, Tourniquets and warmers, Accessories, Position, Neuromuscular Reversal, Transfer patient from the operating room, complications of anesthesia, Seal and signature of the documenting physician and nurse anesthetist). For example, Table 2 shows the data elements of surgery information category. Also, general non-functional requirements of the anesthesia module have been showed in Table 3.

**Table 1:** Demographic information of the patient in the anesthesia module

Demographic data elements
(First Name)
(Last Name)
(Father Name)
(National Code)
(Date of Birth)
(Age)
(Gender)
(Height)
(Weight)
(Medical Record Number)
(Ward)
(Anesthesia Date)
(Admission Date)

**Table 2:** Information elements of surgery information

Surgery data elements
(Preoperative diagnosis)
(Suggested surgery)
(Surgery performed)
Type of Surgery) Elective, outpatient, emergency)
(Surgeon Name)
(Assistant Surgeon Name)
(Anesthesiologist Name)
(Assistant anesthesiologist name)
(Place of surgery)
(Anesthesia nurse name)
(Start of anesthesia)
(Start of operation)
(End of operation)
(End of anesthesia)

**Table 3:** General requirements of the anesthesia module of the operating room department

General requirements of the anesthesia module
While independent, this module is a part of other electronic medical record modules
This module can receive patient identity information from the "Hospital Information System (HIS).”
This module can connect to the "laboratory information system" and receive the laboratory information of the patients who entered the operating room
This module provides access control, accuracy, and validity of the information entered by the user
It is possible to have the same patient file number in the hospital information system and this module
Authorized users can view the patient's previous anesthesia records rec this module
It is possible to access this module by authorized users from outside the department and even from outside the hospital and monitor the residents by the department's attendant
It is possible to read and confirm the recorded information by the anesthesiologist
The details of the anesthesiologist, anesthetist nurse, and the user who accepted the patient, along with the date and time of information registration, can be tracked
It is possible to view the patients' names according to the anesthesiologist’s name
When registering unauthorized data and values, the user is automatically warned
The alphabetical list of the patient's medication history in this module is according to the ATC/DDD system proposed by the World Health Organization
This module can prepare reports at different levels of information

Also, security and privacy requirements such as authentication and verification of users and system protection against cyber-attacks as functional requirements.

### Designing the prototype of the anesthesia module of the electronic medical records in the operating room section of the prayer therapy training center of Shiraz University of Medical Sciences

**Fig. 1:**
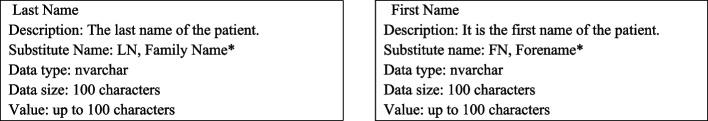
Data dictionary of first name and last name

Data dictionary describe metadata related to each data elements in the system. The data dictionary for the first name provides the description, alternate name, data type, size, and value for the first name data element. (As an example, Fig. 1 shows the data dictionaries of first name and last name).

A scenario describes one way that a system is used, or is envisaged to be used, in the context of an activity in a defined time-frame. In fact, in this study, a scenario has been designed for all non-functional requirements of the anesthesia module. It includes the name of the application, main actors, initial conditions (precondition), flow of activities, final state, and exception conditions. For example, Table 4 shows the scenario of user definition use case in the system.

**Table 4:** User definition scenario in the system

Application name:	User definition in the system
scenario:	User definition in the system
Actors (leading actor):	Anesthesiology assistant (resident), anesthesia superspecialist assistant (fellowship), anesthesiologist, anesthetist nurse
Preliminary conditions:	The system administrator specifies authorized users, and their authentication information is entered
The flow of activities:	The system administrator specifies authorized users and enters their authentication information A unique username and password is defined for each user
Final state:	User registration in the system
Exception conditions:	-

### Implementation of the anesthesia module of electronic medical records in the operating room section of the Namazi Medical Training Center of Shiraz University of Medical Sciences

**Fig. 2:**
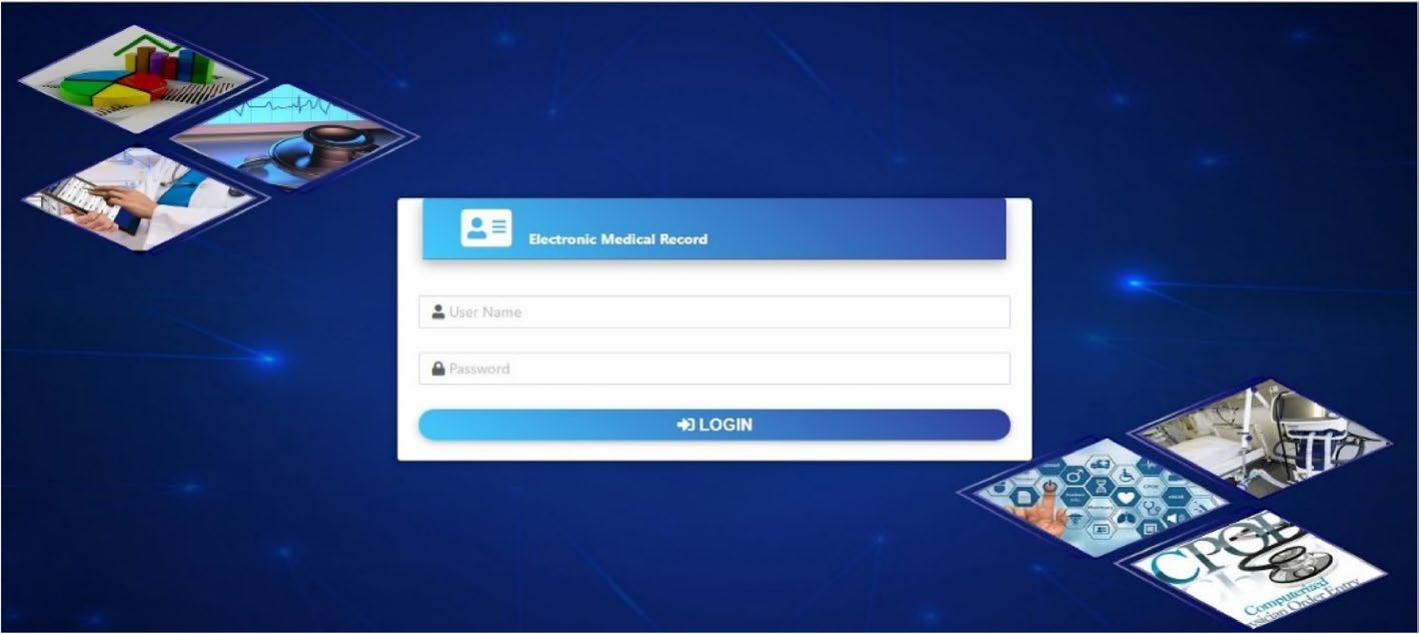
General view of the user entering the module

Hospital Information System (HIS) is active in the operating room department of Namazi Hospital to record medicines and patient information. The prepared module consists of 25 main sections. This module is connected to the Namazi hospital information system (HIS) and laboratory information system (Lab system), which are currently active in Namazi Hospital. Patient identity information is entered into the mentioned module from the hospital information system. In addition, the patient’s laboratory results are automatically entered into the module from the laboratory system. The identity information of patients, including first name, last name, father's name, national code, and admission code, belongs to a hypothetical patient to comply with the principle of patient information confidentiality. Figure 2 shows the general view of user entering the module. Users on this page enter the module with their defined username and password.

**Fig. 3:**
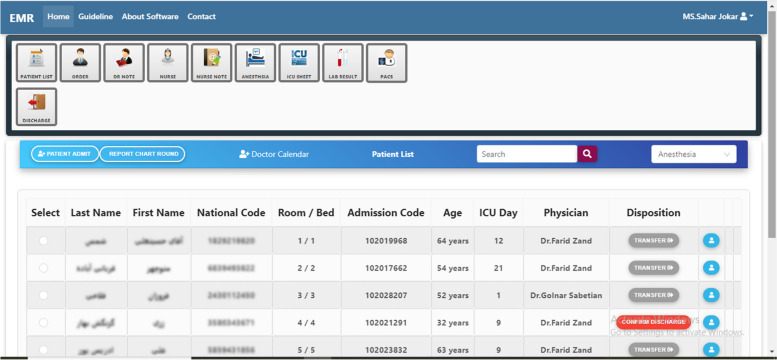
General view of the module

On the general view of the module which has been shown in Fig. 3, the user sees the names of the operating room department, the names of the patients separated by the bed number, the attending physician, and the names of newly admitted patients.

**Fig. 4:**
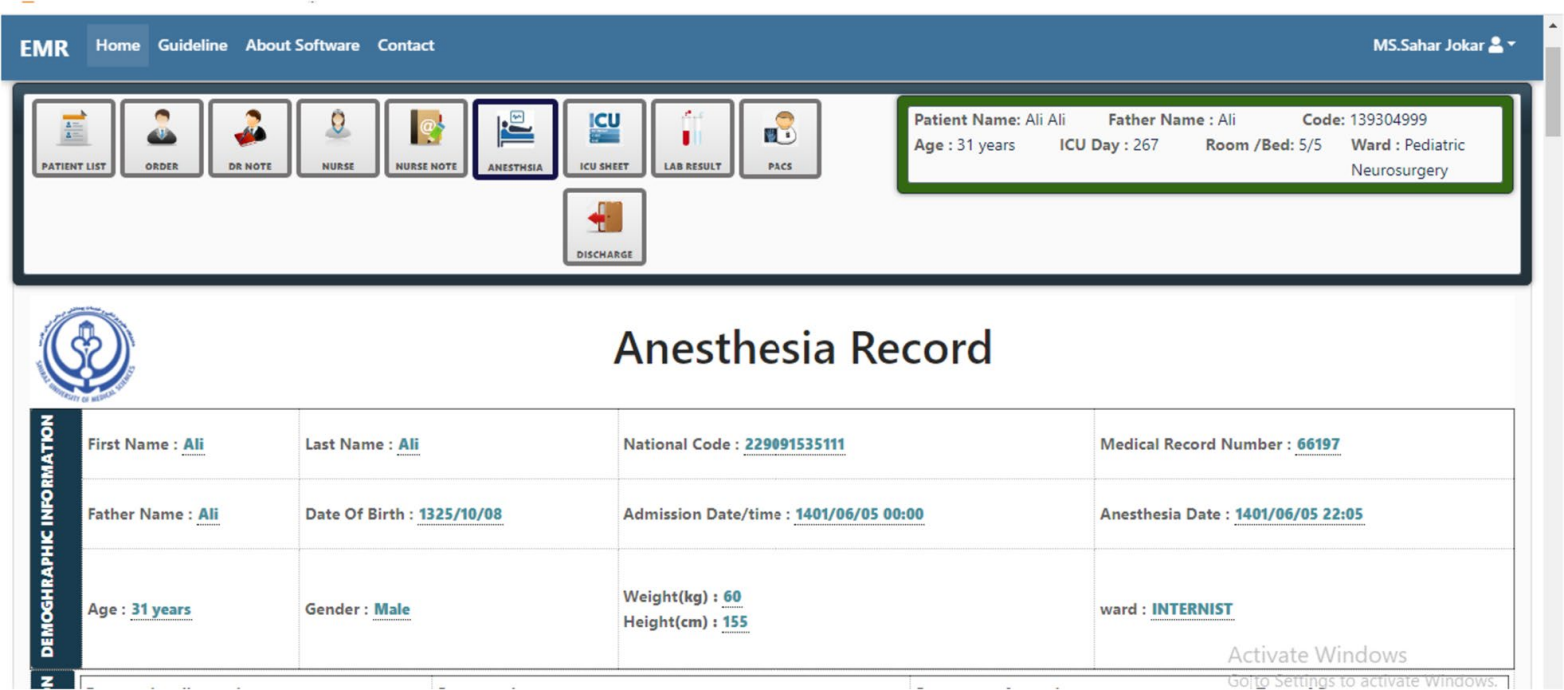
A view of the registration of the patient's identity information

In the Fig. 4, The user can see the patient's identity information on this page, including first name, last name, national code, date of admission, height, weight, gender, etc.

**Fig. 5:**
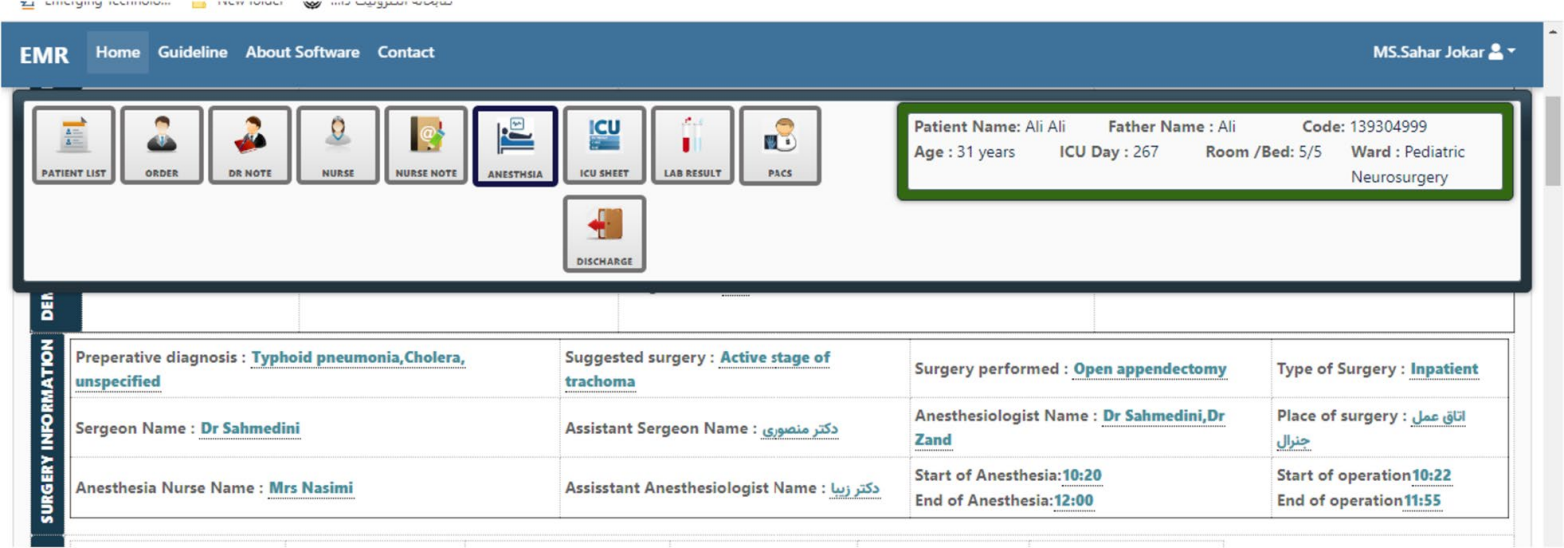
A view of the patient's surgery information record

According to the Fig. 5, the user can view and record the patient's surgery information, including pre-op diagnosis, name of proposed surgery, performed surgery, type of surgery, place of surgery, name of anesthesiologist and surgeon, name of anesthesiologist and surgeon assistants, name of anesthetist nurse, start and end time of operation, and start and end time of anesthesia.

### Evaluation of the usability of the anesthesia module of the electronic medical record in the operating room department of the Namazi Medical Training Center of Shiraz University of Medical Sciences

**Fig. 6:**
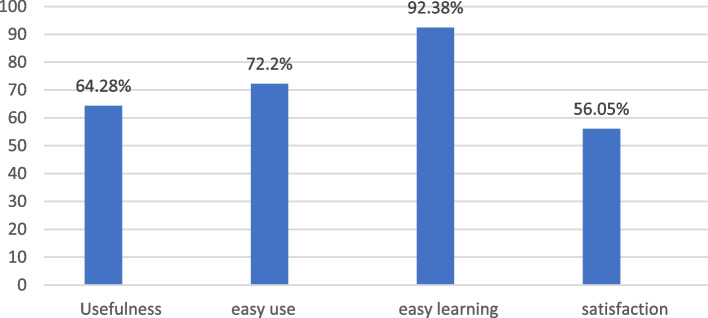
Results of usability evaluation based on USE

The participants in this stage were 3 anesthesiologist assistants and 12 anesthetist nurses. 2 anesthesiologist assistants had less than one year of work experience in the operating room department, and one had between 1 and 2 years of work experience. Anesthetist nurses were in the age range of 28 to 42 years; 66.7% of the evaluated users were female, and 33.3% were male. A total of 94% of users were familiar with ICDL skills, and 6% of them did not have sufficient skills. Figure 6 shows the results of evaluation of the usability of the anesthesia module.

According to Fig. 6, the highest percentage belongs to the easy learning (92.38%) and the lowest percentage belongs to satisfaction. According to the results, on average, 69.1% of users agreed to use this module and find it effectiveness.

## Discussion

Clinical documentation is a fundamental aspect of the encounter between patients and clinical care professionals and its primary purpose, is improving patient care [21, 22], in present study, the functional and non-functional requirements for designing of the module of the electronic medical record in the operating room were determined which is consistent with Sadeghi et al. and Mardani et al. [17, 23]. Information needs to create a standard method for data management, is considered as one of the main factors to achieve efficiency and effectiveness in healthcare systems. The results confirmed general requirements and 286 data elements (identity and clinical information) for the electronic medical record anesthesia module in the operating room department. According to the researcher's investigations, no study has integrated this number of data elements in the field of anesthesia [21–24]. For example. Ahmed et al. (2022) developed recommendations for regional anesthesia in which data elements included patient information, pre-op preparation anesthetic process, central and peripheral nerve block documentation, and procedures after the end of anesthesia [25]. The present study includes nerve block information as well as the places under the nerve block.

In this direction, Sheikh Al-Taifeh et al. (2017) developed 81 structured information elements for an anesthesia information management system used in cesarean sections. These data elements were categorized into eight main groups, including demographic information, pre-operative assessment, type of anesthesia induction, airway management, patient monitoring, medications, fluid intake/output, and post-operative reports. Each category was further divided into subcategories to provide a comprehensive and organized approach to anesthesia information management [26]. In addition to the categories and subcategories already mentioned, the present study also considered other subcategories such as physical examinations, social history, past medical history, and anesthesia history as subgroups in the pre-operative evaluation category. Furthermore, in 2020, the Texas City Children's Hospital launched an anesthesia module in its operating room department, which includes 12 main groups and subgroups. These main groups include demographic information, patient airway, surgical information, intraoperative drugs and fluids, intraoperative airway management, anesthesia chart, intraoperative patient monitoring, anesthesia induction technique, laboratory results, post-operative patient conditions, and information on the anesthesiologists and surgeons involved [27]. All the items mentioned above were considered in the designed module and fully matched in primary groups and sub-groups. The designed module is also more comprehensive compared to the Texas Children's Hospital module, as it includes additional groups such as the person's underlying diseases, family history, history of surgery, anesthesia, and physical examinations.

Electronic medical records increase access to information for clinical, legal and research purposes [28]. One of the data requirements of the medical record is structured data, which has higher accuracy than the unstructured medical record and improves the quality of the collected data. Therefore, using structured data elements is a simple step to improve data quality [29]. Lloyd et al. (1995) and Khorasani et al. (2014) have found that structured forms can be useful in training physicians to write clinical notes. Their studies have shown that using structured forms can facilitate data registration and improve patient training. In situations where time and energy are limited, structured forms can help physicians register information quickly and with minimal written words [30, 31]. The panel of experts in the present study stated that the designed module is structured and leads to accuracy and timely recording of anesthesia data.

In the Canadian Anesthesiology Society's 2020 and 2022 anesthesia guidelines, anesthesia data elements include preparation for anesthesia, airway management information, patient vital signs monitoring, anesthesia chart, patient body temperature management, anesthesia technique, vascular access, nerve blockers, and stimulators [32, 33]. The designed module also includes all data elements mentioned in the guideline. In the meantime, data elements like past medical history, history of family diseases, social history, and the use of patient accessories independently and more widely add to the comprehensiveness of the anesthesia module.

Anesthesia information management systems are integrated with electronic health record modules or independent software and hardware products that have been developed as a means of electronically documenting the details of the patient's anesthesia and physiological status during anesthesia [27]. In this direction, the designed module is also continuously connected with the hospital information systems (HIS) and laboratory information systems (LIS) and receives the required information.

Anesthesiologists are often the first healthcare providers to access patient data history and enter information results. The designed anesthesia module in this study provides complete information about the individual's diseases and family history to the anesthesia providers, which can help to improve continuity of care. Additionally, data entry in anesthesia information management software can enable the creation of a pre-anesthesia assessment that includes required data elements based on the physical status classification of the American Society of Anesthesiologists (ASA) [27].

The designed anesthesia module also allows for the generation of reports based on different parts of anesthesia data, which can be useful for healthcare providers in making treatment decisions. Safdari et al. (2016) and Shahmoradi et al. mentioned that the availability of information in different locations and immediate access to patient records is possible by implementing web-based electronic medical record [18, 34]. The module is web-based, which means that it can be accessed from hospital systems and authorized physician systems outside the hospital for individuals with authorized access. This can help to improve the availability of information in different locations and provide immediate access to patient records, which is important for ensuring high-quality care. Overall, the designed anesthesia module has several advantages that can help to improve the delivery of anesthesia care and enhance patient outcomes.

In present study the usability evaluation of the anesthesia module of the electronic medical record in the operating room was carried out according to the USE standard questionnaire in the four areas of usefulness, ease using, ease of learning, and satisfaction. Features such as usefulness, ease using, and usefulness play an essential role in system usability [35]. Users’ continuous use of a system is determined by their satisfaction and understanding of its usefulness [7, 36]. In this research, 69.1% users agreed with the usefulness, ease of use, learning, and satisfaction of the anesthesia module in the operating room and find it user-friendly, which aligns with Lin's study [37].

One of the limitations of this study is the time it takes to complete the information by users, which can be due to the newness of the launched module. This issue can affect the results of usability evaluation as well. However, it is expected that the speed of recording information will improve over time as users become more familiar with the module and its features. Therefore, the results of periodic evaluations of the anesthesia module in the future are likely to be better and closer to the real results. It is important to continue monitoring and evaluating the effectiveness and usability of the electronic anesthesia module to ensure that it is meeting the needs of healthcare providers and patients.

## Conclusion

With the advancement of medical engineering and anesthesia technologies, the safety of patients requiring anesthesia during surgery has increased. However, the field of anesthesia still poses many risks due to various factors, including medical staff or patient errors and high-risk patients. Therefore, performing safe clinical care depends on obtaining and recording complete and timely information. Developing an electronic anesthesia module can manage a patient's anesthesia information by recording procedures, physiological changes, key events, and drug prescriptions that occur during the pre-, intra-, and postoperative period. In this study, the anesthesia module of the electronic medical record has been implemented in Iran for the first time, due to the necessity of implementing electronic medical records in healthcare centers, especially in the operating room. It is recommended that future studies consider developing other sub-modules of the electronic medical record in the operating room, such as the preoperative evaluation module, surgical procedure description module, and recovery module.

## Data Availability

The datasets generated during the current study are not publicly available. In this study, the minimum dataset which proposed to develop anesthesia module gathered from the medical records of patients at Namazi hospital of Shiraz University of Medical Sciences. To access the dataset, kindly reach out to Dr. Mohammad Ali Sahmeddini, the head of the Adult Surgical Unit at Shiraz University of Medical Sciences. You can contact him at sahmeddini@sums.ac.ir.

## References

[1] Eslami NS, Sardar S, Abbasabadi N. Identification of Effective Factors related to Implementation of Electronic Health Records in Imam Khomeini Hospital, Tehran. Q J Manage Strateg Health Syst. 2020;4(4):337–49.

[2] Bloomfield EL, Feinglass NG (2008). The anesthesia information management system for electronic documentation: what are we waiting for?. J Anesth.

[3] Peterfreund RA, Driscoll WD, Walsh JL, Subramanian A, Anupama S, Weaver M (2011). Evaluation of a mandatory quality assurance data capture in anesthesia: a secure electronic system to capture quality assurance information linked to an automated anesthesia record. Anesth Analg.

[4] Freundlich RE, Barnet CS, Mathis MR, Shanks AM, Tremper KK, Kheterpal S (2013). A randomized trial of automated electronic alerts demonstrating improved reimbursable anesthesia time documentation. J Clin Anesth.

[5] DeCosse M (2020). Delving Deeper into Electronic Medical Records: A Quantitative Methodology Approach to Examining the Influence of Electronic Medical Records on Anesthesiology Provider Satisfaction: Northcentral University.

[6] Bashiri A, Shirdeli M, Niknam F, Naderi S, Zare S (2023). Evaluating the success of Iran Electronic Health Record System (SEPAS) based on the DeLone and McLean model: a cross-sectional descriptive study. BMC Med Inform Decis Mak.

[7] Ahmadi M, Ghazisaeidi M, Bashiri A (2015). Radiology reporting system data exchange with the electronic health record system: a case study in Iran. Global J Health Sci.

[8] Enaizan O, Zaidan A, Alwi NM, Zaidan B, Alsalem M, Albahri O (2020). Electronic medical record systems: decision support examination framework for individual, security and privacy concerns using multi-perspective analysis. Heal Technol.

[9] Nasseri K, Se F (2014). Assessing anesthetic indexes in patient's medical records. Health Inf Manage.

[10] Simpao AF, Rehman MA (2018). Anesthesia information management systems. Anesth Analg.

[11] Rozental O, White RS (2019). Anesthesia information management systems: evolution of the paper anesthetic record to a multisystem electronic medical record network that streamlines perioperative care. J Anesth Hist.

[12] Alkatheri F, Albarrak A, Khan S. Anaesthesia Electronic Records Versus Handwritten Anesthetic Records: An Ambi-directional cohort study. J Health Inform Dev Ctries. 2022;16(1):1–15.

[13] Matava C, Caldeira-Kulbakas M, Chisholm J (2020). Improved difficult airway documentation using structured notes in anesthesia information management systems. Can J Anaesth.

[14] Herasevich V, Ellsworth M, Hebl J, Brown M, Pickering BW (2014). Information needs for the OR and PACU electronic medical record. Appl Clin Inform.

[15] Riahi S, Fischler I, Stuckey MI, Klassen PE, Chen J (2017). The value of electronic medical record implementation in mental health care: a case study. JMIR Med Inform.

[16] Gascón F, Herrera I, Vázquez C, Jiménez P, Jiménez J, Real C (2013). Electronic health record: design and implementation of a lab test request module. Int J Med Informatics.

[17] Mardani H, Bashiri A, Sabetian G, Shokrpour N, Zand F, Masjedi M (2022). Development and evaluation of electronic medical record admission module in intensive care unit: a case study in Iran. Health Managem Inform Sci.

[18] Shahmoradi L, KhoramiMoghadam R, Ghazisaeedi M, Gholamzadeh M (2020). Implementation of electronic health record as a clinical information tool to improve gastric cancer care. Appl Health Inform Technol.

[19] Lapinsky SE (2009). Clinical information systems in the intensive care unit: primum non nocere. Crit Care.

[20] Buchanan EM, Crain SE, Cunningham AL, Johnson HR, Stash H, Papadatou-Pastou M (2021). Getting started creating data dictionaries: how to create a shareable data set. Adv Methods Pract Psychol Sci.

[21] Sandberg WS, Sandberg EH, Seim AR, Anupama S, Ehrenfeld JM, Spring SF (2008). Real-time checking of electronic anesthesia records for documentation errors and automatically text messaging clinicians improves quality of documentation. Anesth Analg.

[22] Schiff GD, Bates DW, Hartzband P, Groopman J, Schiff G (2010). Can electronic clinical documentation help prevent diagnostic errors?. N Engl J Med.

[23] Sadeghi R, Yaghmaei F (2010). The need to review the anesthesia information registration form. J Med Council Islamic Repub Iran.

[24] Karimi S, Saghaeiannejad Isfahani S, Farzandipour M, Esmaeili Ghayoumabadi M. Comparative study of minimum data sets of health information management of organ transplantation in selected countries and presenting appropriate solution for Iran. Health Inform Manage. 2011;7.

[25] Ahmed HM, Atterton BP, Crowe GG, Barratta JL, Johnson M, Viscusi E (2022). Recommendations for effective documentation in regional anesthesia: an expert panel Delphi consensus project. Reg Anesth Pain Med.

[26] Sheykhotayefeh M, Safdari R, Ghazisaeedi M, Khademi SH, Farajolah SSS, Maserat E (2017). Development of a minimum data set (MDS) for C-section anesthesia information management system (AIMS). Anesth Pain Med.

[27] Simpao AF, Rehman MA. Electronic Anesthesia Records: Anesthesia Information Management Systems. Gregory's Pediatric Anesthesia. 6th ed. 2020. p. 1227–1242.

[28] Yeldose A, Dhanyaja N, Jose JT, Anju SS. "Integration of ICU data into electronic medical records-issues and solutions. IEEE Conference on Information & Communication Technologies,Thuckalay, India 2013. 2013:462-7.

[29] Belmin J, de la Fourniere F, Bellot P, Medjahed S, Sibony-Prat J, Bojic N (1998). Quality of the information collected during admission to a hospital geriatric service: importance of a structured medical record. Presse Med.

[30] Goodyear H, Lloyd B (1995). Can admission notes be improved by using preprinted assessment sheets?. BMJ Qual Saf.

[31] Khorasani P, Rassouli M, Zagheri-Tafreshi M, Parvizy S, Nasr EM (2014). Development and evaluation of" Patient Education Record" for structured documentation of patient education process. J Health Promot Manag.

[32] Dobson G, Chow L, Filteau L, Flexman A, Hurdle H, Kurrek M (2020). Guidelines to the practice of anesthesia–revised edition 2020. Can J Anaesth.

[33] Dobson GR (2022). Special announcement-guidelines to the practice of anesthesia-revised edition 2022. Can J Anaesth.

[34] Safdari R, Ghazisaeidi M, Pezeshki A, Mahmoodzadeh B, Nikmaram A (2016). The introduction of electronic medical records for chronic kidney disease as a reliable method for the diagnosis of the disease. J Adv Med Biomed Res.

[35] Adams DA, Nelson RR, Todd PA. Perceived usefulness, ease of use, and usage of information technology: a replication. Princeton, New Jersey: MIS quarterly; 1992. p. 227–47.

[36] Bhattacherjee A. Understanding information systems continuance: an expectation-confirmation model. Princeton, New Jersey: MIS quarterly; 2001. p. 351–70.

[37] Lin H-L, Wu D-C, Cheng S-M, Chen C-J, Wang M-C, Cheng C-A (2020). Association between electronic medical records and healthcare quality. Medicine.

